# A field-acquired RGB–Depth image dataset for computer vision-based baby broccoli detection and size estimation under varying illumination conditions

**DOI:** 10.1016/j.dib.2026.112621

**Published:** 2026-02-21

**Authors:** Rizan Mohamed, Gayan Kahandawa Appuhamillage, Joarder Kamruzzaman, Alexandra Keith, Linh Nguyen

**Affiliations:** a Institute of Innovation Science and Sustainability, Federation University Australia, Northways Road, Churchill, VIC 3842, Australia; b Bulmer Farms, 30 Bulmers Road, Lindenow, VIC 3865, Australia

**Keywords:** Automated harvesting, Crop monitoring, Precision agriculture, Robotic harvesting, Agricultural robotics

## Abstract

This data article describes a curated RGB–Depth image dataset captured using an Intel RealSense D435 stereo depth camera mounted on an autonomous mobile platform during field deployments at commercial baby broccoli farms in Victoria, Australia. The dataset comprises 1759 paired RGB images (640 × 480 pixels) and corresponding 16-bit depth frames acquired under both daytime (natural sunlight) and night-time (LED illumination) conditions, designed to support research in agricultural computer vision and robotic harvesting.

Images were selected from 39,765 raw acquisitions through a reproducible Python curation pipeline applying quality filtering (blur detection, brightness thresholds, corruption detection), perceptual hash-based duplicate removal, and manual review. The final dataset includes 924 daytime and 835 night-time image pairs containing baby broccoli plants at various growth stages. The dataset provides RGB camera intrinsic parameters and pixel-aligned depth maps to enable 3D point cloud reconstruction. Potential applications include developing deep learning models for crop detection and segmentation, validating depth-based size estimation methods, and benchmarking illumination-robust vision systems. All data and curation code are publicly available under a CC BY 4.0 license.

Specifications Table

**Table:** 

Subject	Computer Sciences
Specific subject area	RGB–Depth imaging for agricultural computer vision and robotic crop harvesting
Type of data	Image (RGB and depth maps), Table Raw and curated image files: 8-bit PNG (RGB), 16-bit PNG (depth maps)
Data collection	RGB and depth images were captured using an Intel RealSense D435 camera (640 × 480 pixel resolution, 87° × 58° field of view, 0.2–2.0 m depth range, 50 mm stereo baseline) mounted on a custom autonomous mobile rover during December 2025. Data collection occurred under daytime (natural sunlight) and night-time (LED illumination at 2000–3000 lx) conditions at commercial baby broccoli farms. A reproducible Python curation pipeline reduced 39,765 raw frames to 1759 representative pairs through quality filtering, duplicate removal, and manual review.
Data source location	Institution: Federation University Australia and Bulmer Farms Region: Gippsland, Victoria, Australia Country: Australia Latitude and longitude: Approximately −37.8°S, 147.4°E Collection period: December 2025
Data accessibility	Repository name: Mendeley Data Data identification number: 10.17632/px5p6zdk6k.3 Direct URL to data: https://data.mendeley.com/datasets/px5p6zdk6k/3
Related research article	None

## 1 Value of the Data

• The dataset provides paired RGB and depth images of baby broccoli plants captured under both daytime and night-time conditions, directly supporting development of illumination-robust harvesting systems for real-world operations where baby broccoli is harvested before sunrise (requiring artificial illumination) during summer to preserve freshness, and during both daylight and night-time hours in winter to meet production demands.
• Researchers can use this dataset to develop and evaluate deep learning models for baby broccoli detection and segmentation tasks (using the 1759 image pairs for self-supervised learning or manual annotation), complementing recent advances in RGB–D-based broccoli maturity recognition [1].
• The inclusion of depth information supports development of sensor fusion approaches and 3D spatial understanding algorithms for robotic crop manipulation.
• A subset of 20 image sets with manual ground truth annotations (94 diameter measurements with corresponding depth values) provides a benchmark for validating and testing size estimation algorithms. Note: this ground truth subset is intended for algorithm validation and testing purposes; the sample size is not sufficient for training deep learning regression models.
• The dataset addresses a gap in publicly available RGB–D datasets for specialty vegetable crops, particularly those requiring delicate handling where automated harvesting remains challenging, aligning with the growing role of computer vision and robotics in smart manufacturing systems [2].
• The open-source Python curation pipeline enables full reproducibility and can be adapted for creating similar curated datasets from raw RGB–D acquisitions.

## 2 Background

The dataset was compiled to support research in autonomous harvesting of baby broccoli, where robust perception under varying illumination is critical. RGB–Depth sensing enables 3D understanding of crop geometry for detection and size estimation. Data were collected during real field operations to reflect practical harvesting conditions and to complement prior work on robotic end-effectors [3] and vision-based baby broccoli identification [4].

## 3 Data Description

**Fig. 1:**
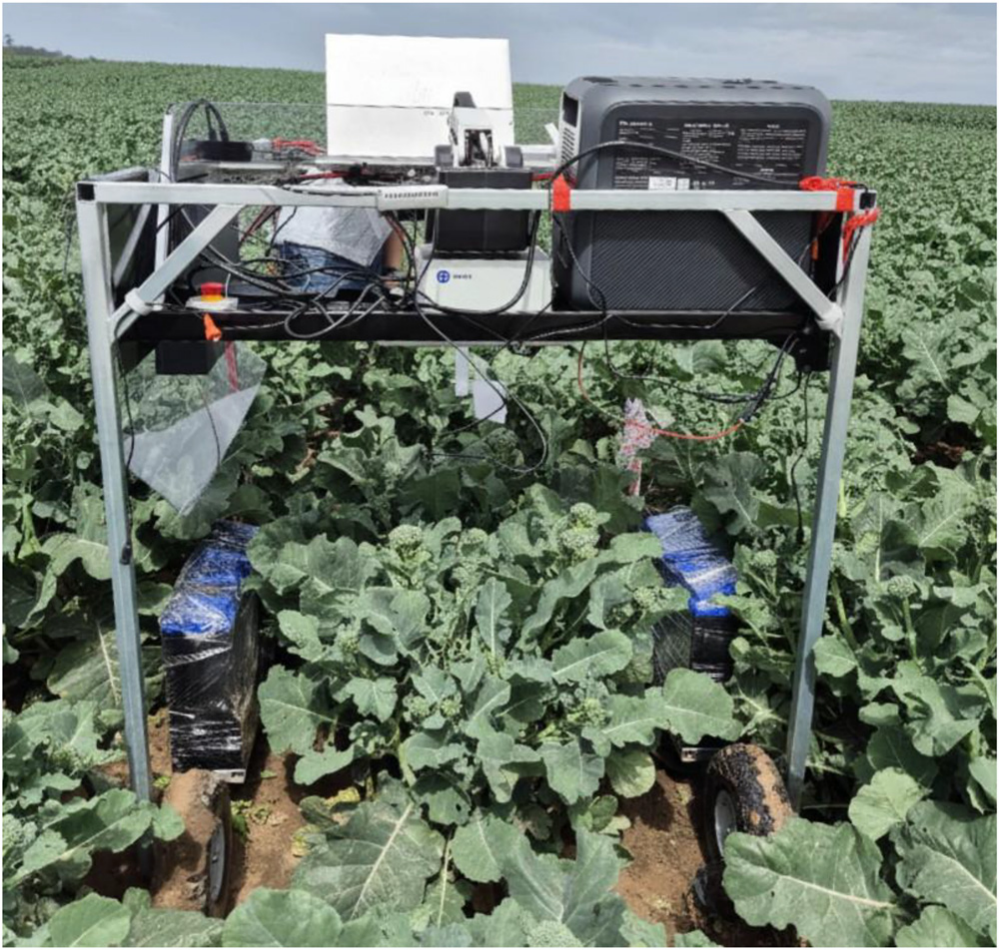
Mobile data collection platform deployed in a commercial baby broccoli field, featuring an Intel RealSense D435 RGB–D camera, portable power station, onboard computing unit, and LED illumination for night-time operation.

The dataset consists of 1759 RGB–D image pairs divided into 924 daytime (52.5 %) and 835 night-time (47.5 %) samples. Fig. 1 shows the robotic rover mobile platform used for data collection.

### 3.1 Dataset organization

Files are organized into a hierarchical folder structure as shown in Table 1[5]. Each RGB image has a corresponding depth image with matching index numbers (e.g., night_rgb_0001.png pairs with night_depth_0001.png).

**Table 1:** Dataset folder structure and specifications.

Folder/File	Description
/nighttime_rgb/	835 night-time RGB images (8-bit PNG, 640 × 480)
/nighttime_depth/	835 night-time depth maps (16-bit PNG, values in mm)
/daytime_rgb/	924 daytime RGB images (8-bit PNG, 640 × 480)
/daytime_depth/	924 daytime depth maps (16-bit PNG, values in mm)
/annotated_dimensions_validation_only/	20 image sets with 94 ground truth diameter annotations (RGB, depth, annotated PNG, JSON metadata)
manual_curation_criteria.txt	Manual curation exclusion and inclusion criteria used during visual review stage
camera_intrinsics.json	RGB camera intrinsic calibration parameters
curate_rgbd_dataset.py	Python curation pipeline script for dataset filtering and selection
rgbd_curation_report.txt	Curation report with parameters and statistics

**Camera:** Intel RealSense D435, 50 mm baseline, 87° × 58° FOV, 0.2–2.0 m range.

Total size: ∼1.1 GB.

The standard PNG format and sequential naming convention ensure direct compatibility with common annotation tools such as CVAT, LabelImg, and Label Studio, enabling researchers to generate bounding boxes or segmentation masks without format conversion.

### 3.2 Camera intrinsics and depth alignment

The dataset includes the RGB camera intrinsic calibration parameters in Table 2 required for converting 2D depth maps into 3D metric point clouds. The intrinsic matrix for the Intel RealSense D435 RGB camera used during data acquisition (at 640 × 480 resolution) is provided in the file camera_intrinsics.json with the following parameters:

**Table 2:** RGB camera intrinsic parameters.

Parameter	Value
Focal length $f_{x}$	612.3895 pixels
Focal length $f_{y}$	611.5399 pixels
Principal point $c_{x}$	331.8402 pixels
Principal point $c_{y}$	252.8850 pixels
Resolution	640 × 480 pixels
Distortion model	Inverse Brown-Conrady

**Important:** All depth maps in this dataset have been aligned (registered) to the RGB camera’s field of view using the Intel RealSense SDK’s rs2::align function. This means the depth and RGB images correspond pixel-for-pixel—the depth value at pixel (u,v) in the depth image corresponds directly to the same pixel location (u,v) in the RGB image. Users can convert any pixel with coordinates (u,v) and depth value Z (in mm) to 3D camera coordinates (X,Y,Z) using the standard pinhole camera model:

$$X=\frac{\left(u-c_{x}\right)\cdot Z}{f_{x}},Y=\frac{\left(v-c_{y}\right)\cdot Z}{f_{y}}$$

### 3.3 Sample images

**Fig. 2:**
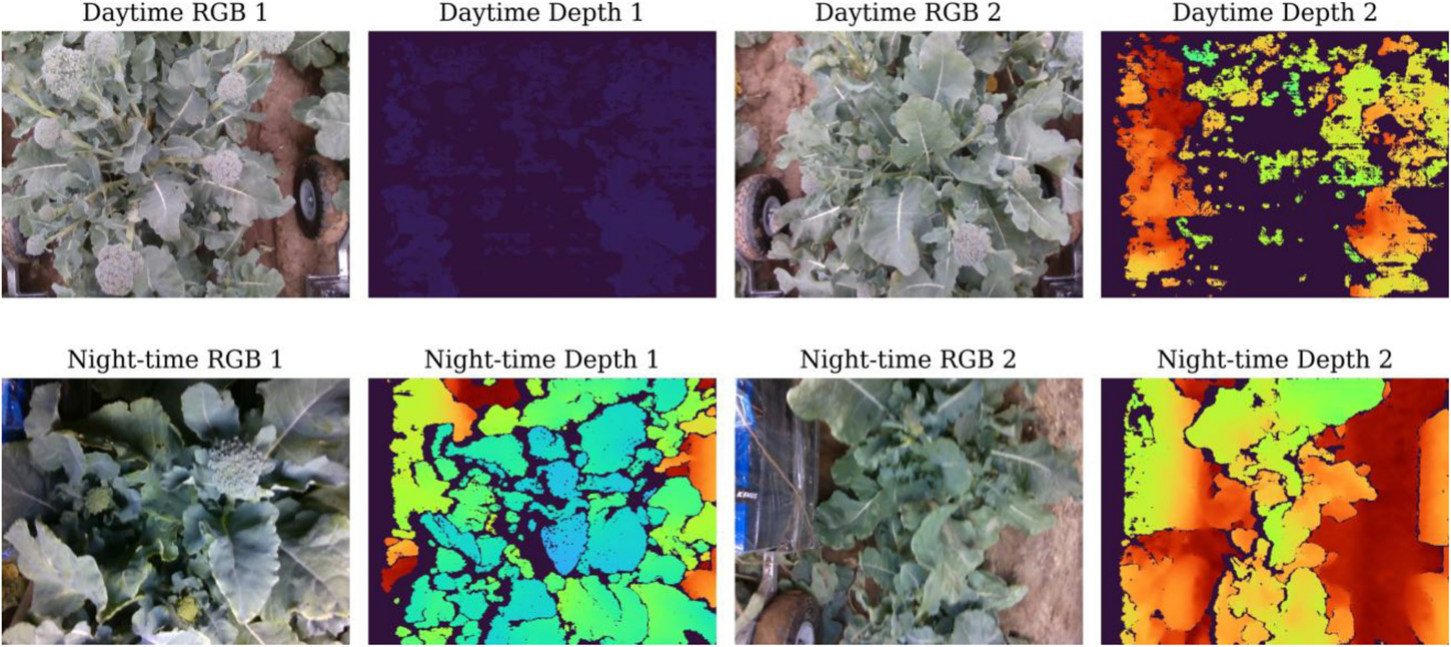
Representative RGB–D samples from the dataset. Top row: daytime acquisitions under natural sunlight showing RGB images and colorized depth maps. Bottom row: night-time acquisitions under artificial LED illumination. Baby broccoli plants at various growth stages are visible with corresponding depth gradients.

Representative RGB–D image pairs are shown in Fig. 2. Daytime images exhibit natural sunlight illumination while night-time images show characteristic artificial LED lighting. Depth maps are colorized using the TURBO colormap where warmer colors indicate closer distances. Note that raw 16-bit depth images appear nearly black when viewed in standard image viewers, as depth values (typically 200–2000 mm) occupy a small portion of the 16-bit range (0–65,535); normalization or colormap application is required for visualization and algorithmic processing.

**Fig. 3:**
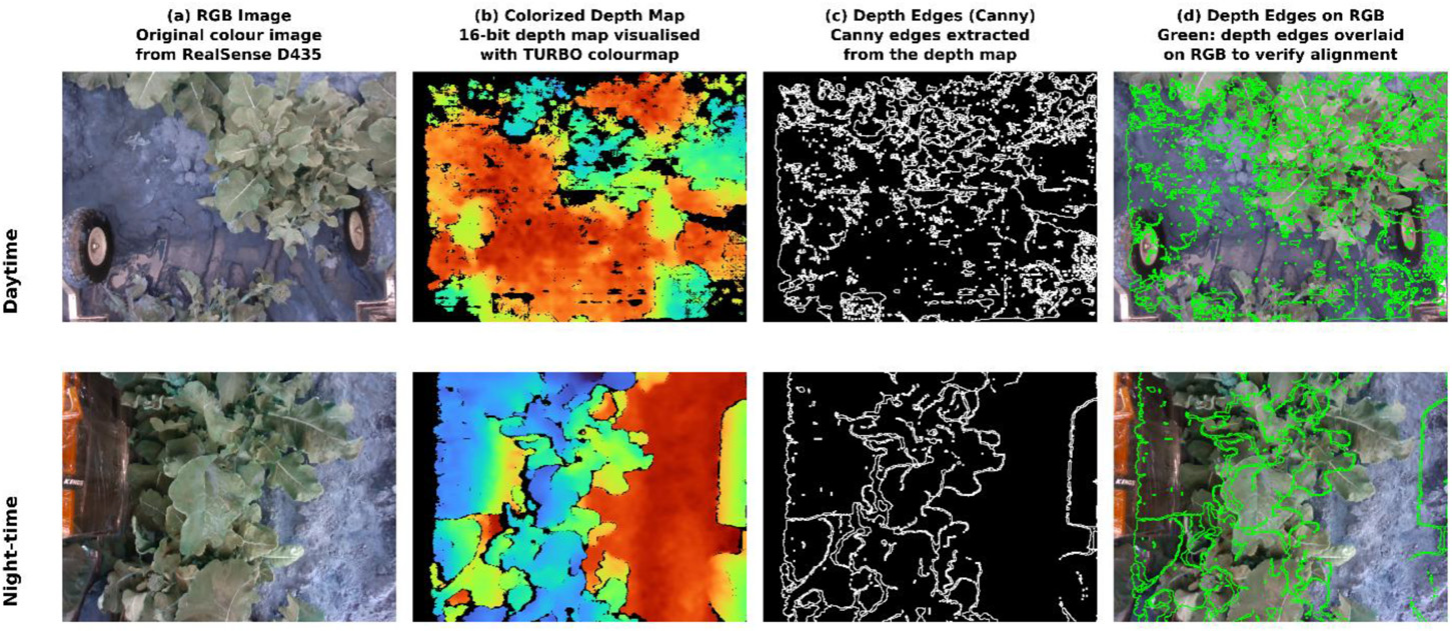
Depth-to-RGB alignment verification for representative daytime (top) and night-time (bottom) image pairs. (a) Original RGB image. (b) Colorized depth map (invalid pixels shown in black). (c) Canny edge map extracted from the depth image. (d) Depth edges (green) overlaid on the RGB image, demonstrating that depth discontinuities align with visible object boundaries, confirming pixel-level registration via the rs2::align function.

To verify the quality of depth-to-RGB alignment, Canny edge maps were extracted from the aligned depth frames and overlaid onto corresponding RGB images. Fig. 3 presents this verification for one daytime and one night-time pair selected for high depth completeness. The overlays confirm that depth edges consistently trace along plant boundaries and structural features visible in the RGB images, with no observable systematic offset.

### 3.4 Ground truth annotations

**Fig. 4:**
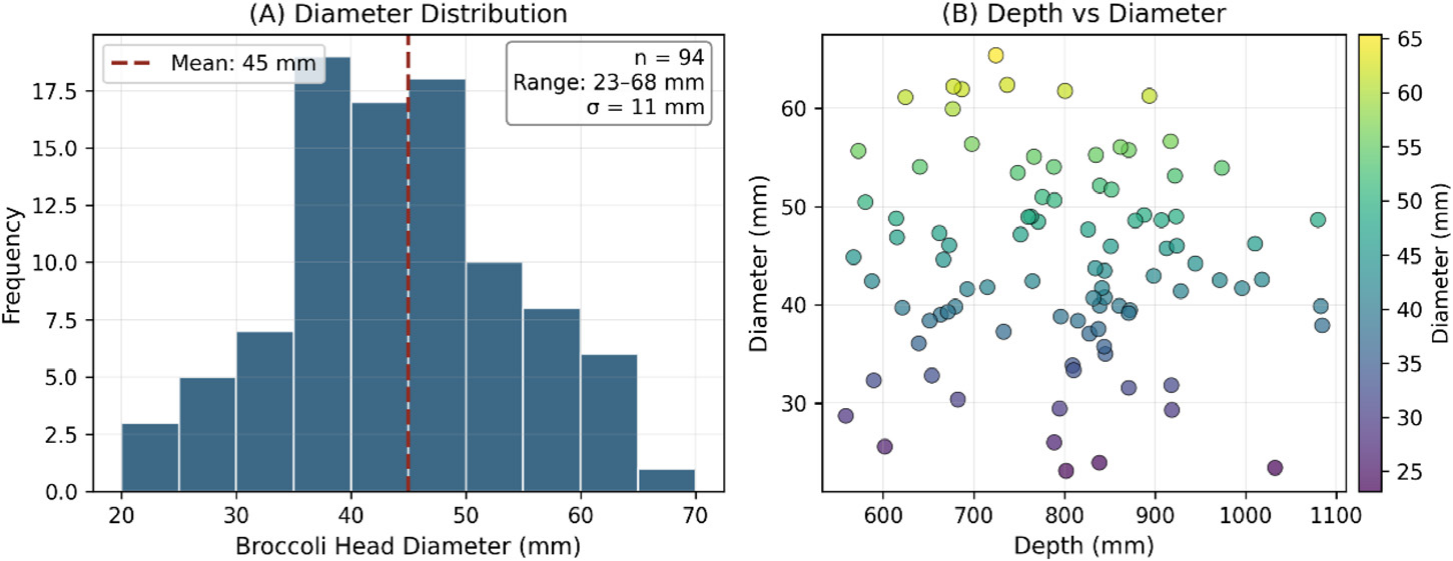
Ground truth annotation summary. (A) Distribution of manually measured broccoli head diameters showing range of 23–68 mm with mean 45 mm. (B) Scatter plot illustrating measurement coverage across the operational depth range (554–1087 mm).

A subset of 20 image sets includes manual ground truth annotations for broccoli head diameter estimation, comprising 94 point measurements with pixel coordinates, measured diameters (mm), and corresponding depth values (mm). Fig. 4 summarizes the annotation characteristics. Each annotated set contains four files: clean RGB image, depth map, annotated visualization, and JSON metadata with measurement details.

Table 3 summarizes the ground truth annotation subset characteristics.

**Table 3:** Ground truth annotation summary statistics.

Parameter	Value
Image sets	20
Total annotations	94
Annotations/image	3–5
Diameter range	23–68 mm
Diameter mean ± SD	45 ± 11 mm
Depth range	554–1087 mm
Depth mean	800 mm
Image resolution	640 × 480 px
Format	PNG + JSON

### 3.5 Quality metrics

**Fig. 5:**
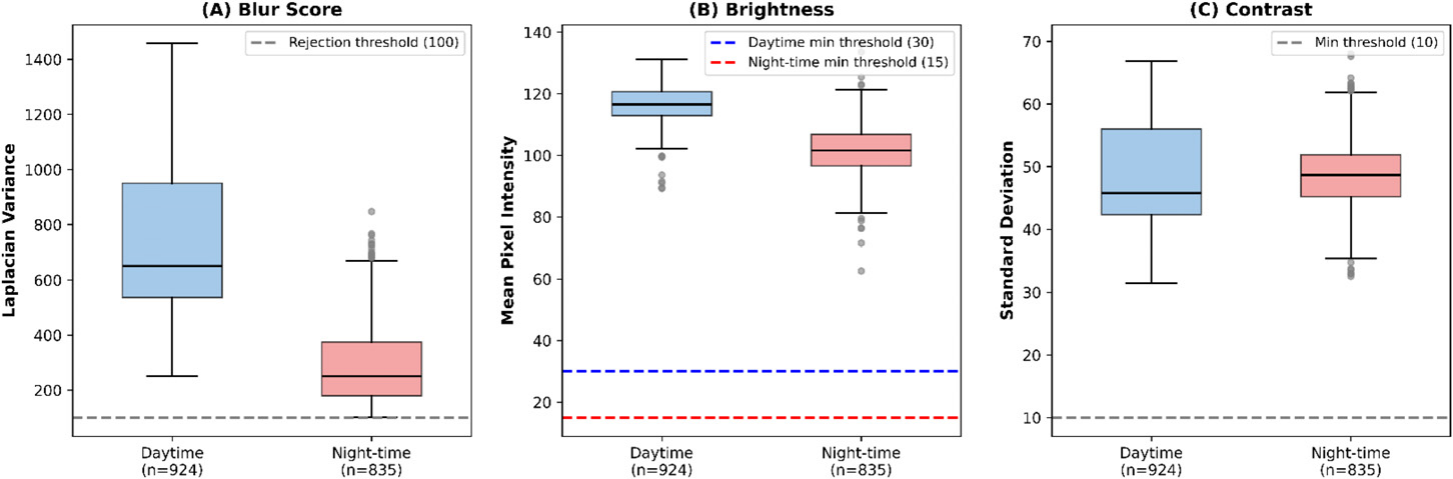
Box plot comparison of quality metrics between daytime and night-time images in the curated dataset. (A) Blur score measured as Laplacian variance—higher values indicate sharper images; the grey dashed line indicates the rejection threshold of 100, below which images were discarded as blurry. (B) Mean pixel intensity (brightness); dashed lines indicate minimum brightness thresholds: 15 for night-time images (red) and 30 for daytime images (blue), accommodating the inherently lower brightness of LED-illuminated night-time acquisitions. (C) Image contrast measured as standard deviation of pixel intensities. The grey dashed line indicates the minimum contrast threshold of 10.

Fig. 5 presents the distribution of image quality metrics between daytime and night-time subsets. Daytime images exhibit higher blur scores (mean Laplacian variance: 721.4 vs. 291.2), indicating sharper images likely due to better illumination enabling shorter exposure times. Night-time images show lower brightness (mean intensity: 102.2 vs. 116.5) but comparable contrast (std. deviation: 48.7 vs. 46.9), illustrating that LED illumination provides adequate scene visibility for image capture. Depth measurements cluster between 0.3–1.5 m, consistent with the camera mounting height (∼0.8 m) and crop canopy structure.

At the operational depth range of 0.5–1.1 m, the D435′s 50 mm stereo baseline yields a theoretical depth error of approximately ±2–6 mm, which is within acceptable tolerance for broccoli head size estimation (diameters: 23–68 mm).

Depth map completeness is summarised in Table 4 across both illumination conditions. Daytime images exhibit substantially higher rates of invalid (zero-valued) depth pixels (mean: 62.2 %) compared to night-time images (mean: 24.1 %). This disparity is consistent with well-documented infrared interference from direct sunlight on structured-light stereo sensors. No depth-specific post-processing filters were applied during capture or curation, preserving raw depth measurements for researchers to apply their own filtering or completion strategies. Despite the higher invalid pixel rates in daytime frames, regions of primary interest — broccoli heads at operating distances of 0.3–1.0 m — generally retain valid depth values, as the sensor performs best at close range on diffuse, textured surfaces.

**Table 4:** Depth completeness comparison between daytime and night-time subsets.

Metric	Daytime (*n* = 924)	Nighttime (*n* = 835)
Mean invalid pixels ( %)	62.2	24.1
Median invalid pixels ( %)	60.9	23.8
Std. deviation ( %)	11.5	4.8
Min ( %)	28.0	13.2
Max ( %)	88.6	41.2

## 4 Experimental Design, Materials and Methods

### 4.1 Data acquisition

RGB–D sensing combines color imagery with depth information to enable 3D perception for agricultural robotics applications [6]. Data were acquired using an Intel RealSense D435 RGB–D camera, which has been successfully deployed in various agricultural monitoring tasks [7], mounted on a custom autonomous mobile rover (Fig. 1). The platform comprised an aluminum-frame wheeled chassis, portable lithium power station (1000 Wh), onboard computing unit running Ubuntu, and adjustable LED array for night-time illumination. The camera was mounted at approximately 0.8 m height, oriented downward toward the crop canopy.

Data collection was conducted at Bulmer Farms in Gippsland, Victoria, Australia during December 2025. Daytime collection occurred under natural sunlight, while night-time collection used LED illumination (2000–3000 lx) after sunset. Multiple acquisition sessions, each defined as a single rover traversal of one crop row, yielded 39,765 raw RGB–D pairs (33,823 night-time from 5 row traversals; 5942 daytime from 2 row traversals conducted in late morning and late afternoon to capture varying solar angles).

### 4.2 Ground truth annotation

Ground truth diameter measurements were collected for a subset of 20 image sets to enable validation of automated size estimation algorithms. The annotation process employed a two-stage methodology combining digital marking with physical measurement to ensure accuracy.

A custom annotation software tool was developed in Python to facilitate precise marking of broccoli head locations in RGB images. The software displays synchronized RGB and depth image pairs, allowing annotators to click on individual broccoli heads to record pixel coordinates. For each marked point, the software automatically extracts the corresponding depth value from the aligned depth map and stores annotations in structured JSON format including pixel coordinates (x, y), depth value (mm), and a unique identifier for each measurement.

Physical diameter measurements were obtained using a digital vernier caliper (±0.01 mm resolution) during field data collection sessions. For each annotated broccoli head, the maximum crown diameter was measured by positioning the caliper jaws at the widest point of the floret head. Measurements were recorded immediately following image capture to minimize temporal variation due to plant growth or environmental changes. Each image set contains 3–5 annotated broccoli heads, yielding 94 total diameter measurements across the 20 annotated sets.

The resulting annotations provide paired ground truth data linking image-space coordinates with physical measurements, enabling researchers to develop and validate depth-based size estimation algorithms. The annotation software source code is included in the repository to support reproducibility and adaptation for similar annotation tasks.

### 4.3 Data curation pipeline

**Fig. 6:**
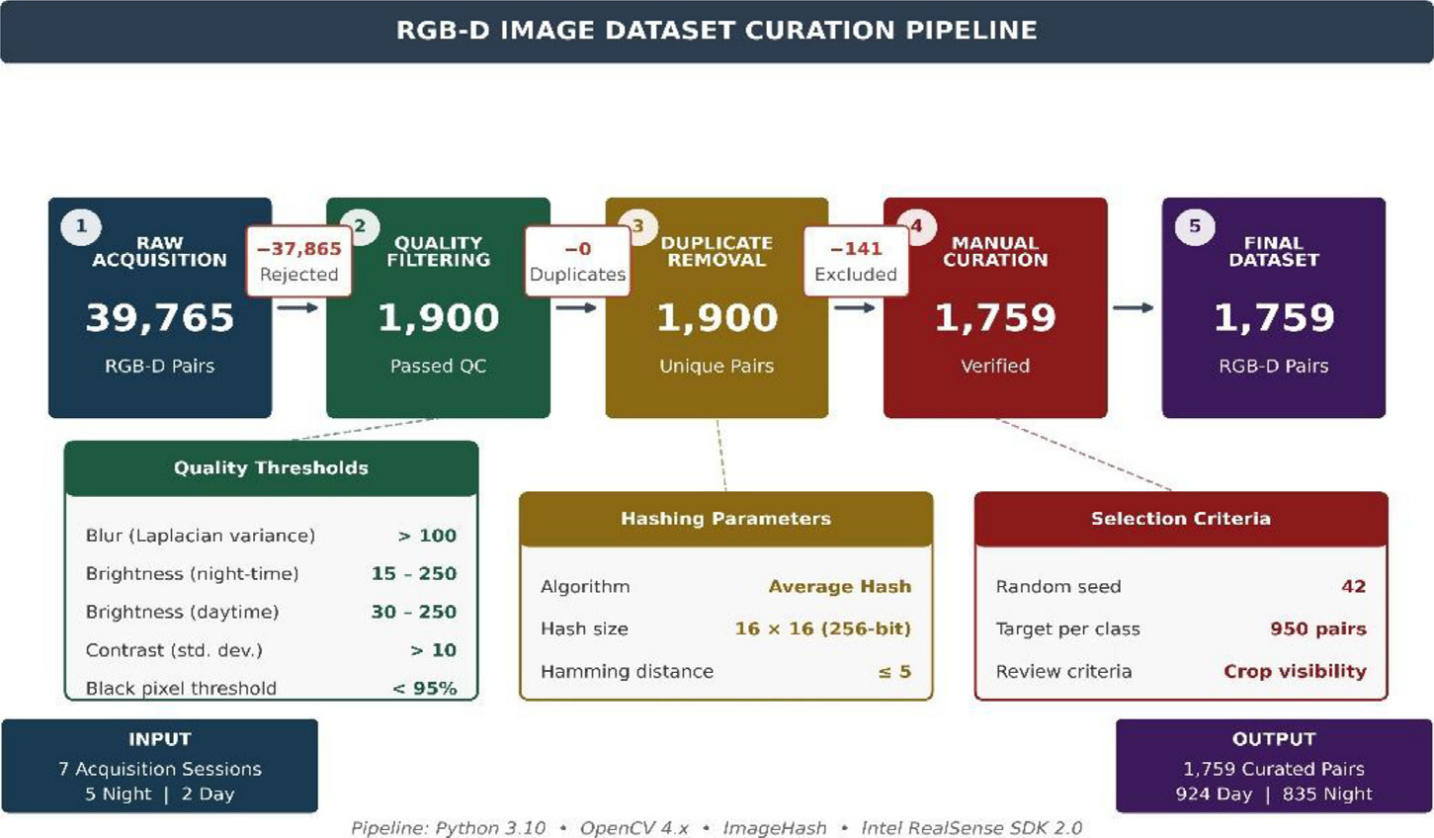
Data curation pipeline flowchart showing progressive filtering from 39,765 raw acquisitions to 1759 final image pairs.

A Python-based curation pipeline was developed to select high-quality, non-redundant image pairs. The pipeline in Fig. 6 is provided as open-source code for full reproducibility. The high reduction ratio (39,765 to 1759 pairs) reflects the continuous frame capture during slow platform traversal, resulting in substantial near-duplicate imagery that was systematically removed to ensure dataset diversity.

The pipeline performs four stages:

(1) **Quality filtering**—images are assessed for blur (Laplacian variance *>*100), brightness (15–250 for night-time, 30–250 for daytime), contrast (std. dev. *>*10), and corruption (*<*95 % black pixels)
(2) **Duplicate removal**—perceptual hashing (16 × 16 average hash) identifies near-duplicates (Hamming distance ≤5), retaining one per cluster
(3) **Random selection**—950 pairs per illumination condition selected using fixed seed (42)
(4) **Manual curation**—visual inspection removes frames with insufficient broccoli content or quality issues. Manual review (first author reviewed, supervisors validated) was limited to exclusion of frames with insufficient crop visibility or severe acquisition artefacts and did not involve subjective labeling or interpretation. Table 5 summarizes the curation results.

**Table 5:** Data curation pipeline results.

Stage	Criteria	Pairs
Raw acquisition	5 night + 2 day sessions	39,765
Quality filtering	Blur, brightness, contrast, corruption	*>*20,000
Duplicate removal	Perceptual hash (threshold=5)	*>*10,000
Random selection	950 night + 950 day (seed=42)	1900
Manual curation	Visual quality review	1759
**Final dataset**	**924 daytime + 835 night-time**	**1759**

## Limitations

The dataset reflects conditions at Gippsland-region farms in southeastern Australia and may not generalize to other geographic regions or cultivation practices. Data were collected during a single growing season (December 2025); seasonal variations and different cultivars are not captured. The RealSense D435 depth sensor exhibits reduced accuracy on thin structures, specular surfaces, and at range extremes; daytime images are particularly affected, with an average of 62.2 % invalid depth pixels compared to 24.1 % for night-time images due to infrared interference from sunlight. The dataset does not include pixel-level annotations; researchers requiring segmentation masks or bounding boxes must generate them independently. All daytime data were collected under clear sky conditions on a single day; weather variation (e.g., overcast vs sunny) is not represented. The primary focus of this dataset is providing illumination-diverse RGB-D imagery for training detection models; the ground truth diameter annotations (94 measurements across 20 image sets) were collected specifically for validating size estimation algorithms rather than for training regression models.

## Ethics statement

The authors have read and complied with the ethical requirements for publication in Data in Brief. This work does not involve human participants, animal experiments, or social media data. Data collection occurred on private land with permission from Bulmer Farms. Images containing incidental capture of farm workers were excluded during curation.

## CRediT Author Statement

**Rizan Mohamed**: Conceptualization, Methodology, Software, Data curation, Writing – original draft, Writing – review & editing. **Gayan Kahandawa Appuhamillage**: Supervision, Methodology, Writing – review & editing. **Joarder Kamruzzaman**: Supervision, Writing – review & editing. **Alexandra Keith**: Resources, Investigation, Writing – review & editing. **Linh Nguyen**: Supervision, Writing – review & editing.

## Data Availability

(Mendeley Data).Field-Acquired RGB-Depth Image Dataset for Baby Broccoli Detection and Size Estimation Under Varying Illumination Conditions (Original data)
